# Histone 3 Hyperacetylation and the Aggressive Behavior of Giant Cell Lesions

**DOI:** 10.1002/cam4.71785

**Published:** 2026-04-05

**Authors:** Caio César da Silva Barros, Éricka Janine Dantas da Silveira, Márcia Cristina da Costa Miguel, Rogerio Moraes Castilho, Cristiane Helena Squarize

**Affiliations:** ^1^ Epithelial Biology Laboratory, Department of Periodontics and Oral Medicine University of Michigan School of Dentistry Ann Arbor Michigan USA; ^2^ Postgraduate Program in Dental Sciences, Oral Pathology and Medicine, Department of Dentistry Federal University of Rio Grande Do Norte Natal RN Brazil

**Keywords:** Giant cell granuloma, Giant cells, histone, multinuclear cells

## Abstract

**Introduction:**

Giant Cell Lesions exhibit variable aggressive clinical behavior. Understanding the molecular mechanisms of these lesions can facilitate a more personalized and effective therapeutic approach.

**Material and Methods:**

The acetylation of Histone H3 at Lysine 9 (H3K9ac) and the expression of Inhibitor of Growth Protein 5 (ING5) were evaluated in 19 cases of Peripheral Giant Cell Granuloma (PGCG), 19 cases of non‐aggressive Central Giant Cell Granuloma (CGCG), 19 cases of aggressive CGCG, and 19 cases of Giant Cell Tumor of Bone (GCTB), totaling 76 cases of Giant Cell Lesions.

**Results:**

H3K9 hyperacetylation was found in aggressive Giant Cell Lesions compared to non‐aggressive lesions (*p <* 0.05). Aggressive Giant Cell Lesions also show higher ING5 expression in multinucleated giant cells and cannibalistic multinucleated giant cells compared to non‐aggressive lesions (*p <* 0.05). There was no difference in the levels of H3K9ac and ING5 between aggressive Central Giant Cell Granuloma and Giant Cell Tumor of Bone (*p* > 0.05). H3K9ac and ING5 were associated with aggressive characteristics in the CGCG (*p <* 0.05).

**Conclusion:**

H3K9 hyperacetylation highlights the significance of this epigenetic event in the aggressiveness of Giant Cell Lesions and may indicate their potential for aggressive behavior, thereby providing information to improve treatment strategies, particularly for Central Giant Cell Granuloma. Understanding the CGCG's clinical behavior is essential for determining the therapeutic modality and avoiding long‐term post‐treatment sequelae.

## Graphical abstract

Acetylation of Histone H3 at Lysine 9 (H3K9) was determined in Peripheral Giant Cell Granuloma (PGCG), Non‐aggressive Central Giant Cell Granuloma (NAg CGCG), Aggressive Central Giant Cell Granuloma (Ag CGCG), and Giant Cell Tumor of Bone (GCTB). H3K9 hyperacetylation correlated with the aggressive behavior of Giant Cell Lesions. Furthermore, H3K9 hyperacetylation is associated with aggressive clinical and radiographic features such as symptomatology, growth rate, tooth displacement, root resorption, and cortical bone perforation in Central Giant Cell Granuloma.

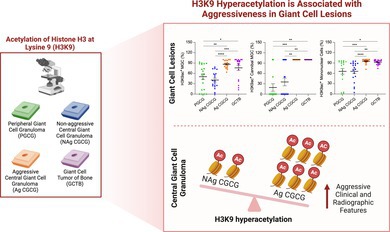

**Key Abbreviations**
(Ag CGCG): Aggressive CGCG
(CGCG): Central Giant Cell Granuloma
(CMGC): Cannibal Multinucleated Giant Cells
(GCTB): Giant Cell Tumor of Bone
(MC): Mononuclear cells
(MGC): Multinucleated Giant Cells
(NAg CGCG): Non‐aggressive CGCG
(PGCG): Peripheral Giant Cell Granuloma

## 1 Introduction

Central Giant Cell Granuloma (CGCG), also known as Giant Cell Lesions of the jaws, is an osteolytic lesion characterized by mononuclear cell proliferation and the presence of multinucleated giant cells (MGC). CGCG exhibits histopathological similarity with Peripheral Giant Cell Granuloma (PGCG) and Giant Cell Tumor of Bone (GCTB). However, CGCG exhibits variable clinical behavior, whereas PGCG and GCTB are indolent and aggressive, respectively [1, 2]. CGCG's nature is still unknown, and no clinicopathological characteristics or markers make it possible to predict its clinical behavior accurately. Thus, understanding the molecular mechanisms related to its pathogenesis and aggressiveness can help establish a more personalized and effective therapeutic approach [3, 4].

Cell cannibalism is the process by which a cell engulfs and digests other cells. It has been linked to increased cellular survival, invasion, and metastasis in neoplasms, thereby promoting more aggressive biological behavior [1, 2, 5]. Although it is still uncertain how the CGCG's histopathological characteristics contribute to the prediction of its clinical behavior, morphological studies indicate an association between the number of cannibalistic multinucleated giant cells (CMGC) and the clinical and radiological features of aggressiveness in this lesion, such as growth rate, tooth displacement, and tooth root resorption [1, 2, 5]. Additionally, it has been demonstrated that autophagy is induced in osteoclast precursor cells simultaneously with osteoclastogenesis, and it is believed that autophagy and cell cannibalism share the same molecular mechanism [6, 7].

Histone modifications are epigenetic dynamic events that regulate chromatin organization and gene transcription from embryogenesis to tissue homeostasis and disease. These epigenetic events are often related to the acetylation of lysine residues in the tails of histones 3 and 4 [8, 9, 10]. The acetylation process occurs due to the action of histone acetyltransferase enzymes. In this context, the Inhibitor of Growth Protein 5 (ING5) is a component of the MOZ/MORF complex, which presents acetyltransferase activity and promotes acetylation of histones H3 Lysine 9 (H3K9ac) during osteoclastogenesis [11, 12, 13]. To date, no studies have investigated the correlation between H3K9ac/ING5 activation and clinical aggressiveness in CGCG and its different cellular subpopulations, especially CMGCs. Here, we investigated the immunohistochemical expression patterns of H3K9ac and ING5 in Giant Cell Lesions, their association with cell cannibalism, and the clinical behavior of these lesions.

## 2 Material and Methods

### 2.1 Study Design

This retrospective and cross‐sectional research was approved by the Research Ethics Committee of the Federal University of Rio Grande do Norte (approval #5,632,269). The sample comprised 19 cases of PGCG, 19 cases of non‐aggressive CGCG, and 19 cases of aggressive CGCG from the Laboratory of Oral Pathology, Department of Dentistry, Federal University of Rio Grande do Norte, as well as 19 cases of GCTB provided by the Getúlio Sales Diagnostics laboratory. All eligible cases diagnosed between 1990 and 2018 were reviewed. For each giant cell lesion group, consecutive cases were included, ensuring that selection was based on predefined inclusion and exclusion criteria. It included cases of CGCG with clinical and radiographic information for their clinical classification. PGCG, CGCG, and GCTB with adequate/available formalin‐fixed paraffin‐embedded tissue to carry out the study. In contrast, cases of CGCG and GCTB that underwent treatment before the lesion surgical excision and patients diagnosed with cherubism, hyperparathyroidism, or syndromes associated with Giant Cell Lesions were excluded from the study. Clinical and radiographic data related to symptomatology, growth rate, teeth displacement, root resorption, and cortical bone perforation were collected in the biopsy request forms to perform CGCG clinical classification as non‐aggressive (NAg) and aggressive (Ag) as proposed by Choung et al. [14]. The growth rate was considered fast when the lesion showed prominent growth in three to six months [14].

### 2.2 Immunohistochemistry

Three‐μm‐thick sections were obtained from paraffin‐embedded tissue blocks and mounted on organosilane‐coated slides for the immunohistochemical study (3‐aminopropyltriethoxysilane slides; Sigma Chemical Co., USA). Initially, tissue deparaffinization and rehydration were performed, followed by antigenic recovery (10 mM citric acid; pH 6.0) and endogenous peroxidase blockade. Subsequently, the sections were washed in PBS and incubated with 1% bovine serum albumin (BSA in PBS). Then, specimens were incubated with the primary antibodies: anti‐H3K9ac (clone C5B11; Cell Signaling Technology, USA; dilution 1:400, overnight) and anti‐ING5 (clone sc‐2025; Santa Cruz Biotechnology, USA; dilution 1:200, overnight), followed by the secondary antibodies. The reactions were revealed using 3,3′‐diaminobenzidine (DAB; Sigma‐Aldrich, USA), resulting in a brown stain. Sections were counterstained with hematoxylin QS (H‐3404; Vector Laboratories, USA). Histological sections of giant cell tumor and inflammatory fibrous hyperplasia were used as positive controls for primary anti‐H3K9ac and anti‐ING5 antibodies, respectively. The primary antibody was replaced with 1% BSA for the negative control.

### 2.3 Immunohistochemical Assessment

For each slide, three independent fields (ROIs) were photographed in areas showing microscopic evidence of the lesion, especially those with higher cellularity. Within these regions, multiple fields were selected to ensure adequate representation of the three cellular subpopulations (mononuclear cells, MGC, and CMGC). Selection was made independently of immunostaining intensity, prior to evaluating the staining, to avoid sampling bias. Photos were taken using the Leica CTR5000 light microscope (Leica Microsystems; USA) coupled with the QImaging MicroPublisher camera 6 (model 01‐MP6‐R‐CLR‐14‐C; Teledyne QImaging; USA) at ×200 magnification. Cell cannibalism phenomenon in MGCs was identified morphologically based on the criteria established by Sarode et al. [1].: (1) “partial” cell cannibalism was considered when the CMGC presented pseudopod formation toward a smaller cell, while (2) “complete” cell cannibalism was characterized by a smaller cell present within the CMGC, where a clear halo surrounded this smaller cell. Mononuclear cells, MGC, and CMGC that exhibited brownish nuclear and/or cytoplasmic staining were considered positive. Furthermore, nuclear and cytoplasmic immunoreactivity was analyzed separately.

Quantitative analysis of mononuclear cells, MGC, and CMGC for H3K9ac and ING5 was performed by a blinded single examiner using a computer‐assisted analysis with a standardized algorithm in QuPath (version 0.5.0) to ensure reproducible and objective scoring across all samples. Cells were classified as positive or negative based on the algorithm's parameter, with a threshold of 10 (brownish stain: Positive: > 10; Negative: < 10). Quantitative analyses for both H3K9ac and ING5 were calculated through the labeling index to the percentage of cells stained for the marker: (number of positive cells/total number of counted cells) x 100 [8]. ING5 scores were also determined among the mononuclear cells, classified as Score 0 (negative immunoexpression); Score 1 (< 25% of positive cells); Score 2 (between 25% and 50% of positive cells); Score 3 (between 50% and 75% of positive cells); Score 4 (> 75% of positive cells).

### 2.4 Statistical Analysis

Data were analyzed using the GraphPad Prism software (GraphPad Prism 10.2.2, San Diego, CA, USA). The Shapiro–Wilk test was used to evaluate whether a data set is normally distributed. The Kruskal–Wallis test and post hoc Dunn's test were performed to determine differences between the expression of H3K9ac and ING5 in the lesions. Mann–Whitney test was used to evaluate the association between this expression and the clinical and radiographic characteristics (symptomology, growth, tooth displacement and/or root resorption, and perforation of cortical bone) of CGCGs. Spearman's correlation coefficient was used to verify correlations between H3K9ac and ING5 expression. Values of *p ≤* 0.05 were considered statistically significant. Asterisks denote statistical significance (*p ≤ 0.05; **p ≤ 0.01; ***p ≤ 0.001; ****p ≤ 0.0001). Data were expressed as mean ± standard error of the mean (SEM).

## 3 Results

### 3.1 Hyperactivation of H3K9 Is Correlated With Aggressive Behavior of Giant Cell Lesions

**FIGURE 1:**
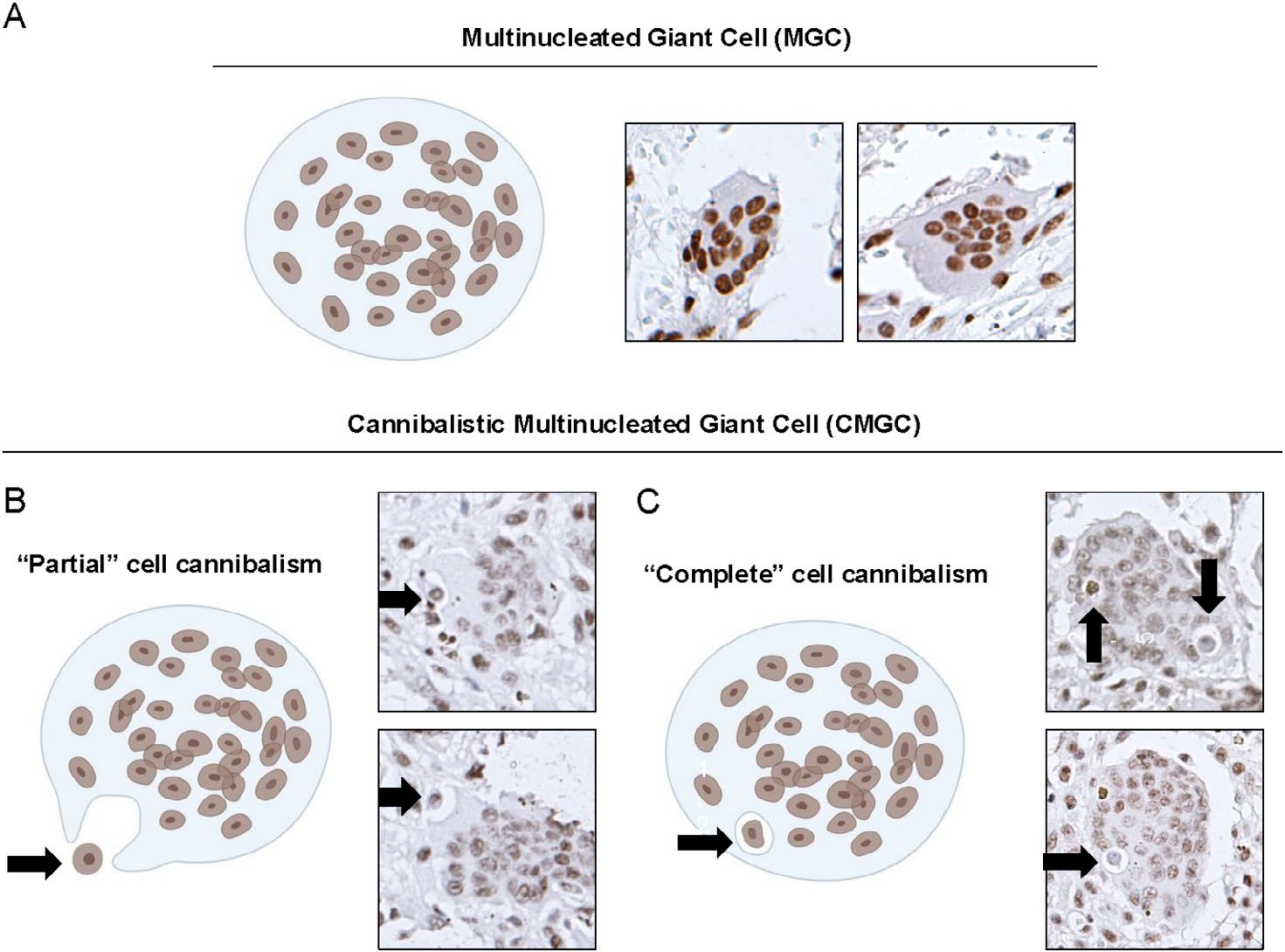
Cell cannibalism analysis in giant cell lesions. Morphological representation of a typical (A) multinucleated giant cell (MGC) and (B–C) cannibalistic multinucleated giant cells (CMGC). (B) “Partial” cell cannibalism was noted when the CMGC extended pseudopods toward a smaller cell. Conversely, (C) “Complete” cell cannibalism was identified by a smaller cell surrounded by a clear halo inside the CMGC. (Immunostaining for H3K9ac in Central Giant Cell Granuloma cases; Magnification: ×200).

**FIGURE 2:**
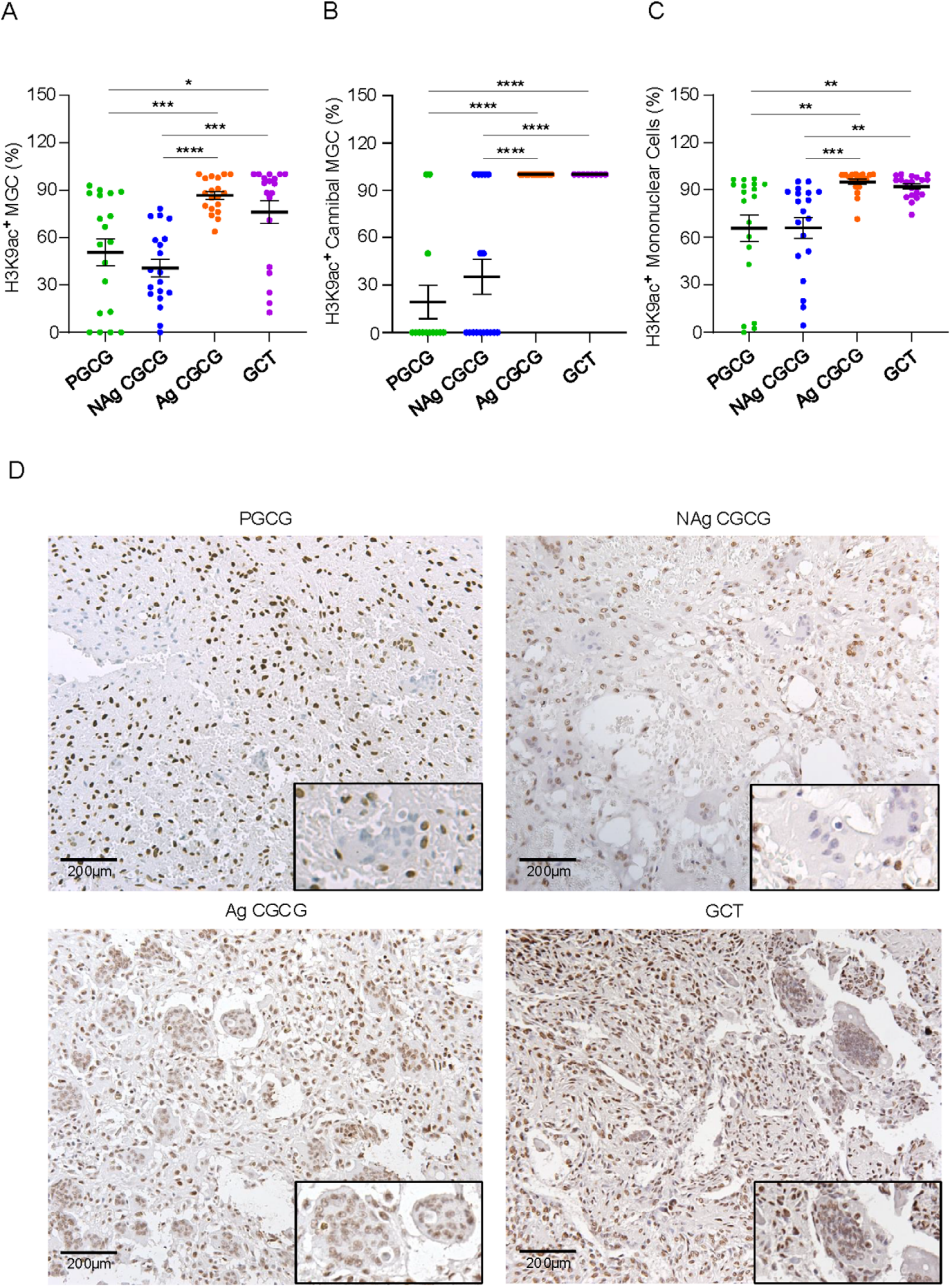
Histone H3 lysine 9 acetylation (H3K9ac) expression in Giant Cell Lesions. H3K9ac expression levels are similar between Peripheral Giant Cell Granuloma (PGCG) and non‐aggressive Central Giant Cell Granuloma (NAg CGCG), as well as between aggressive Central Giant Cell Granuloma (Ag CGCG) and giant cell tumor (GCTB) of bone, in (A) mononuclear cells, (B) multinucleated giant cells (MGC), and (C) cannibal multinucleated giant cells (CMGC) (*p ≤ 0.05; **p ≤ 0.01; ***p ≤ 0.001; ****p ≤ 0.0001) (Data represented as mean ± SEM). (D) Representative images of H3K9ac expression in PGCG, NAg CGCG, Ag CGCG, and GCTB. Inset: H3K9ac expression in CMGC (Scale bars: 200 μm).

Giant Cell Lesions display diverse cell populations, including MGC, which exhibit a cannibalistic phenomenon known as CMCG. The morphological features of a typical MGC and CMGC are shown in Figure 1A–C. Specifically, partial cell cannibalism was observed when the CMGC extended pseudopods toward a smaller cell (Figure 1B). In contrast, complete cell cannibalism was identified by a smaller cell surrounded by a clear halo within the CMGC (Figure 1C). Upon analyzing the expression in different cell populations (i.e., MGC, CMGC, mononuclear cells) that comprise the Giant Cell Lesions, we observed that hyperacetylation of H3K9 in the nucleus was significantly greater in the aggressive (Ag) Giant Cell Lesions (Figure 2). Indeed, MGC of Ag CGCG showed hyperacetylation of H3K9 (86.6% ± 2.4) compared to non‐aggressive (NAg) CGCG (40.8% ± 5.5; ****p < 0.0001) and PGCG (50.5% ± 8.3; **p < 0.0052). The same pattern was found on GCTB (76.1% ± 7.2), which was also hyperacetylated compared to PGCG (*p = 0.02) and NAg CGCG (***p < 0.0007) (Figure 2A). Similar results were observed when looking at the acetylation of H3K9 in the CMGC of the Giant Cell Lesions. CMGC in the Ag CGCG (100% ± 0) exhibited higher H3K9ac nuclear expression compared to PGCG (19.2% ± 10.6; ***p < 0.0002) and NAg CGCG (35.2% ± 11.1; **p < 0.0021) as well as GCTB (100% ± 0) compared to PGCG (**p < 0.0012) and NAg CGCG (**p < 0.0099) (Figure 2B). Regarding the mononuclear cells, Ag CGCG (95% ± 1.6) and GCTB (92.1% ± 1.6) exhibited significant hyperacetylation of H3K9 compared to PGCG (65.7% ± 8.4; ***p = 0.0007 and *p = 0.01, respectively) and NAg CGCG (65.9% ± 6.6; ****p < 0.0001 and **p = 0.0053, respectively) (Figure 2C). No statistical differences existed in H3K9ac expression between nonaggressive lesions, such as PGCG and NAg CGCG. In testing, aggressive lesions (CGCG and GCTB) also displayed similar levels of hyperacetylation of H3K9 (Figure 2A–C). All cell types showed clear nuclear staining for H3K9, with a complete absence of cytoplasm in Giant Cell Lesions (Figure 2D).

The correlation results revealed that H3K9ac expression was positively and significantly correlated between mononuclear cells and MGC (ρ = 0.682; ****p < 0.0001) in NAg CGCGs, and a positive and significant correlation in H3K9ac expression between mononuclear cells and MGC (ρ = 0.682; *p =* **0.001) in Ag CGCGs (Figure S1).

### 3.2 ING5 Upregulation Is Found in Aggressive Giant Cell Lesions

**FIGURE 3:**
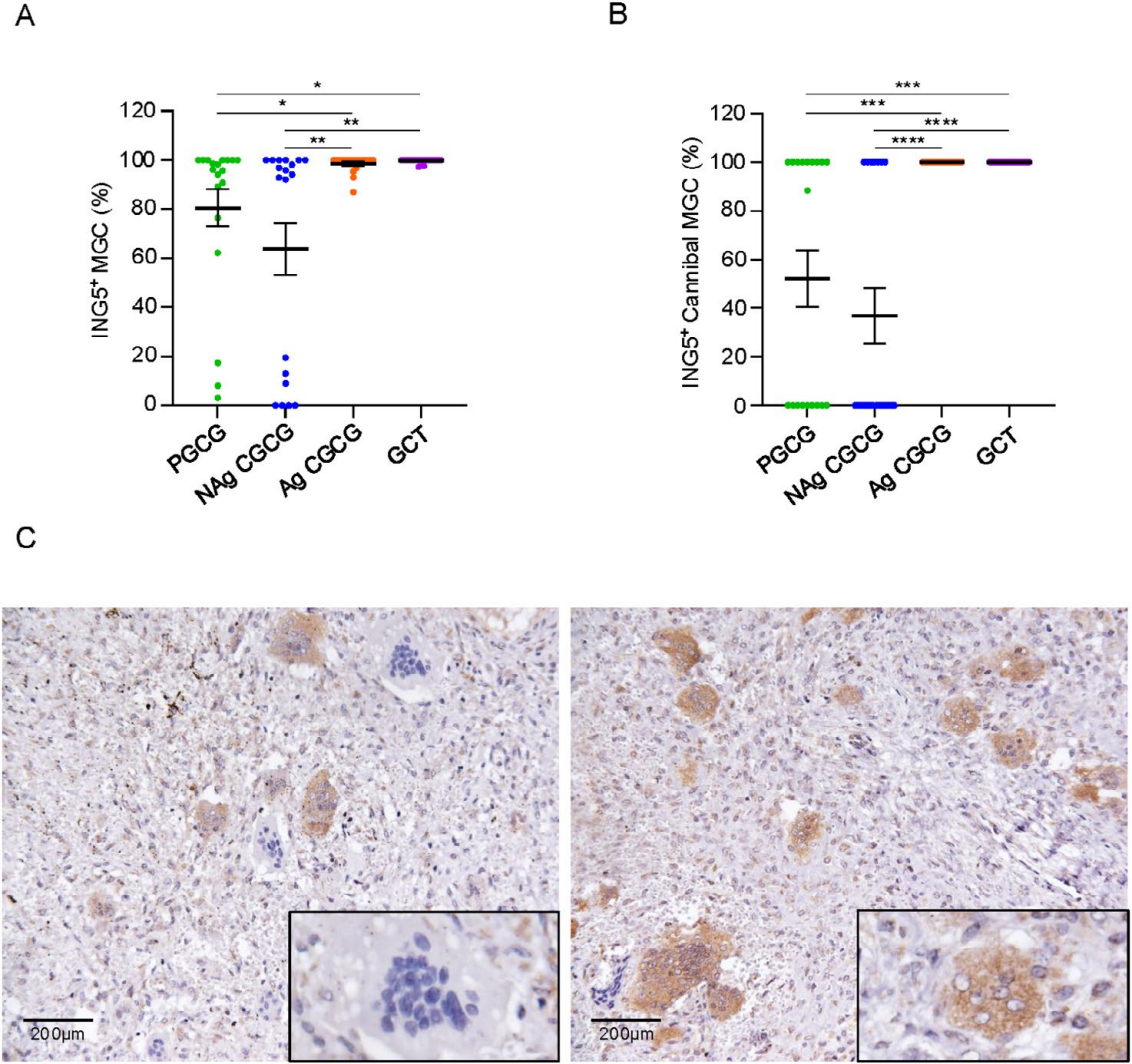
ING5 expression in multinucleated giant cells (MGC) and cannibal multinucleated giant cells (CMGC) of Giant Cell Lesions. (A) ING5 expression was significantly higher in MGC in aggressive Central Giant Cell Granuloma (Ag CGCG) and giant cell tumor (GCTB) of bone compared to Peripheral Giant Cell Granuloma (PGCG) and non‐aggressive Central Giant Cell Granuloma (NAg CGCG). (B) CGMC from Ag CGCG and GCTB exhibited a significantly higher ING5 expression than PGCG and NAg CGCG (*p ≤ 0.05; **p ≤ 0.01; ***p ≤ 0.001; ****p ≤ 0.0001) (Data represented as mean ± SEM). (C) Representative images of ING5 expression in MGC. Note the positive cytoplasmic immunostaining only in cells that present nuclei with vesicular morphology (Scale bars: 200 μm).

**FIGURE 4:**
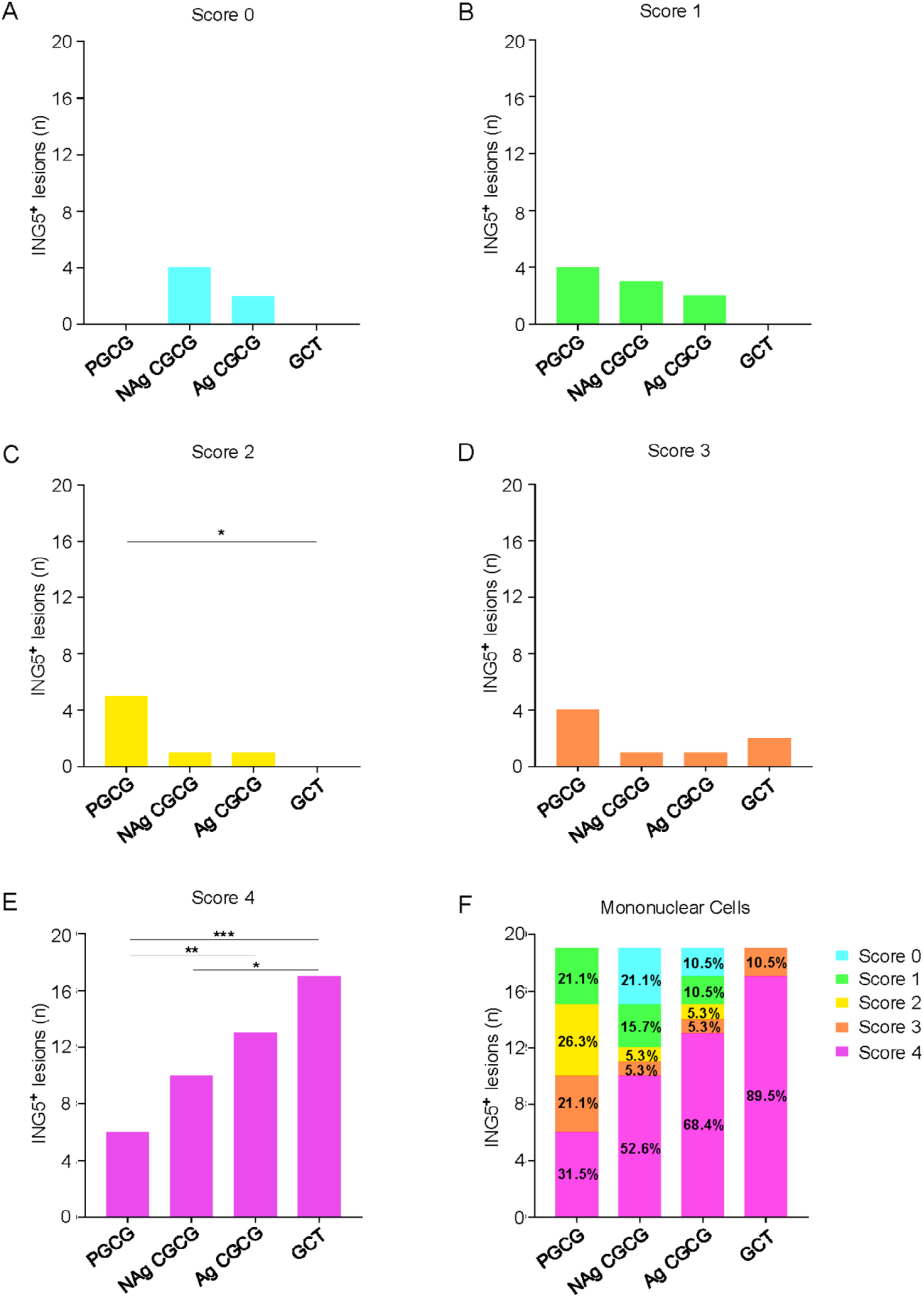
ING5 expression in mononuclear cells of Giant Cell Lesions. (A) Only non‐aggressive Central Giant Cell Granuloma (NAg CGCG) and aggressive Central Giant Cell Granuloma (Ag CGCG) showed cases with negative expression (Score 0) of ING5. (B) Peripheral Giant Cell Granuloma (PGCG) exhibited a higher frequency of Score 1 (< 25% positive cells). (C) PGCG presented a significantly higher frequency of Score 2 (25% to 50% positive cells) than giant cell tumor (GCTB) of bone. (D) PGCG and GCTB exhibited a higher frequency of Score 3 (50% to 75% positive cells) compared to NAg CGCG and Ag CGCG. (E) A significantly higher frequency of Score 4 (> 75% positive cells) was observed in GCTB compared to PGCG and NAg CGCG, as well as in Ag CGCG compared to PGCG. (F) Frequency distribution of ING5 expression scores in PGCG, NAg CGCG, Ag CGCG, and GCTB.

ING5 was present in the cytoplasm of the cells, particularly in the multinucleated cells (i.e., MGC and CMGC) (Figure 3A–C). In non‐aggressive lesions, the PGCG (80.5% ± 7.5) and NAg CGCG (63.7% ± 10.4) exhibited fewer numbers of MGC with ING5 (ING5^+^) compared to Ag CGCG (98.5% ± 0.7) (*p = 0.0101; **p = 0.001, respectively) and the GCTB (99.6% ± 0.1) (**p = 0.0019; ***p = 0.0001, respectively) (Figure 3A). Similarly, PGCG (52% ± 11.6) and NAg CGCG (36.8% ± 11.3) displayed less ING5^+^ CMGC compared to Ag CGCG (100% ± 0) (**p = 0.0029; ****p < 0.0001, respectively) and GCTB (100% ± 0) (**p = 0.0029; ****p < 0.0001, respectively) (Figure 3B). Interestingly, across all lesions, MGC and CMGC with ING5 exhibited vesicular nuclei, whereas MGC and CMGC lacking ING5 exhibited hyperchromatic nuclei (Figure 3C). Additionally, the ING5 immunostaining profile in Giant Cell Lesions showed predominantly cytoplasmic ING5 expression in the mononuclear cells. We observed a significantly higher frequency of score 4 in GCTB compared to NAg CGCG and PGCG (*p = 0.0293; ***p = 0.0002, respectively) and in Ag CGCG compared to PGCG (**p = 0.0086) (Figure 4E). In summary, GCTB exhibited the highest frequency of score 4 (89.5%), followed by the Ag CGCG (68.4%), NAg CGCG (52.6%), and PGCG (31.5%) (Figure 4F). The correlation results revealed a positive and significant correlation in ING5 expression between mononuclear cells and MGC (ρ = 0.573; **p = 0.008) in Ag CGCGs (Figure S1).

### 3.3 H3K9 Hyperacetylation and ING5 Upregulation Are Associated With Clinical and Radiographic Features of Aggressiveness in Central Giant Cell Granuloma

**FIGURE 5:**
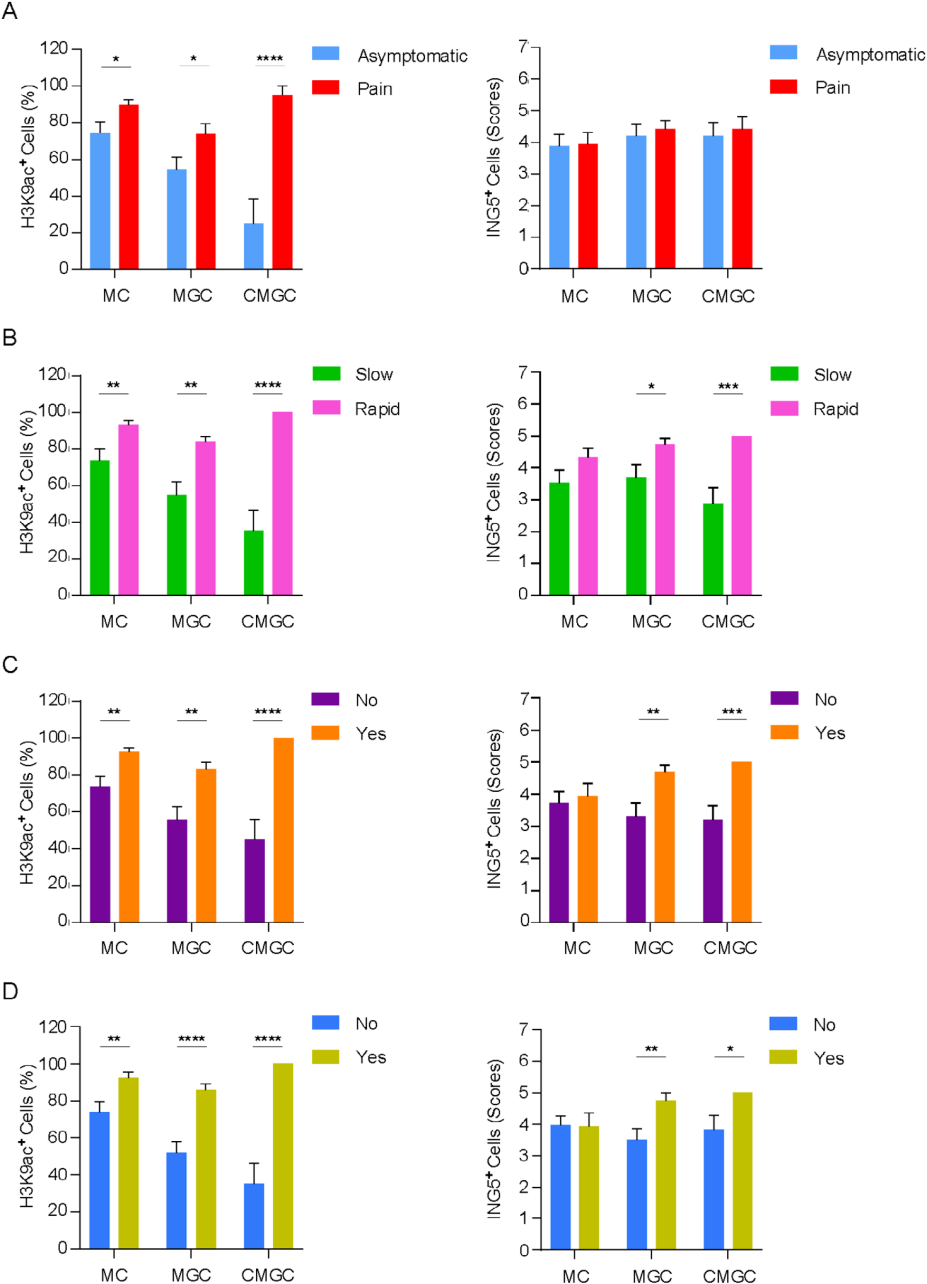
Histone H3 lysine 9 acetylation (H3K9ac) and ING5 expression in mononuclear cells (MC), multinucleated giant cells (MGC), and cannibal multinucleated giant cells (CMGC) of Central Giant Cell Granulomas (CGCG) and its association with clinical and radiographic features. (A) H3K9ac expression was significantly higher in MC, MGC, and CMGC of symptomatic CGCG. (B) CGCG, exhibiting rapid clinical growth, showed significant H3K9 hyperacetylation in MC, MGC, and CMGC, as well as higher ING5 expression in MGC and CMGC. The presence of (C) tooth displacement and/or root resorption and (D) cortical bone perforation was significantly associated with H3K9 hyperacetylation in MC, MGC, and CMGC, and ING5 upregulation in MGC and CMGC (*p ≤ 0.05; **p ≤ 0.01; ***p ≤ 0.001; ****p ≤ 0.0001) (Data represented as mean ± SEM).

Next, we determine whether H3K9ac and ING5 were correlated with aggressive clinical and radiographic parameters of Central Giant Cell Granulomas, as shown in Figure 5. Quantitative analysis revealed that mononuclear cells (89.7% ± 2.9; *p = 0.033), MGC (74% ± 5.6; *p = 0.0343), and CMGC (96.4% ± 3.5; ****p < 0.0001) exhibited significant H3K9 hyperacetylation in symptomatic CGCG (Figure 5A). Additionally, it was observed a significantly H3K9 hyperacetylation in mononuclear cells (73.6% ± 6.2; **p = 0.0038), MGC (54.7% ± 7.2; **p = 0.0033), and CMGC (35.2% ± 11.1; ***p = 0.0002), as well as ING5 expression in MGC (3.6% ± 0.4; *p = 0.0305) and CMGC (2.8% ± 0.4; **p = 0.0033), in lesions that exhibited rapid clinical growth (Figure 5B).

Regarding the presence of tooth displacement and/or root resorption, there was a significantly H3K9 hyperacetylation in mononuclear cells (92.3% ± 2.4; *p = 0.0281), MGC (83.3% ± 3.5; *p = 0.0386), and CMGC (100% ± 0; **p = 0.0049), while ING5 expression was significantly higher in MGC (4.6% ± 0.2; *p = 0.0137) and CMGC (5% ± 0; *p = 0.027) in CGCG that exhibited these radiographic characteristics (Figure 5C). Similarly, CGCG that exhibited cortical bone perforation showed significantly H3K9 hyperacetylation in mononuclear cells (92.3% ± 3.1; *p = 0.0253), MGC (85.8% ± 3.3; ***p = 0.0004), and CMGC (100% ± 0; ***p = 0.0002), as well as ING5 expression in MGC (4.7% ± 0.2; *p = 0.0367) (Figure 5D). Thus.

Overall, our results suggest that Giant Cell Lesions with similar clinical behavior have similar expression patterns of H3K9 acetylation. Aggressive Giant Cell Lesions, in particular, exhibit H3K9 hyperacetylation. Moreover, the higher expression of H3K9ac and ING5 is associated with clinical and radiographic characteristics of aggressiveness of the Central Giant Cell Granuloma.

## 4 Discussion

CGCG of the jaws is a benign lesion with variable and sometimes aggressive clinical behavior, with a propensity to recur after treatment. Understanding the CGCG's clinical behavior is essential to determine the therapeutic modality and avoid long‐term post‐treatment sequelae. Although there is a clinical classification system for this lesion, it is still difficult to predict which CGCG will display aggressive behavior [5, 15]. Here, we revealed changes in the H3K9 acetylation status in Giant Cell Lesions with different clinical behaviors and demonstrated that the H3K9ac profile contrasts according to CGCG's non‐aggressive and aggressive clinical behavior.

Gene expression dysregulation through histone acetylation and deacetylation has been associated with several pathologies. In bone tissue, basal H3K9 acetylation has been associated with the expression of genes linked to osteogenic differentiation, whereas hyperacetylation promotes higher expression of RANK‐L and osteoclastogenesis through activation of the non‐canonical Wnt pathway [9, 13, 16, 17]. In the context of Giant Cell Lesions, it is known that GCTB harbors H3F3A mutations, which may be related to H3K9 acetylation [4]. Although the CGCG is unlikely to carry this mutation [18], our results show a similar pattern of H3K9ac between aggressive CGCG and GCTB, indicating that H3K9 hyperacetylation may be a landmark molecular feature in Giant Cell Lesions that exhibit aggressive clinical behavior.

We demonstrated here that the higher acetylation of H3K9 is associated with the growth, tooth displacement, root resorption, and cortical bone perforation of aggressive CGCG. These results highlight the importance of this epigenetic event in the differentiation and clastic activity of the MGC. Our results also revealed an association between H3K9ac and pain. Notably, it has been demonstrated that calcitonin gene‐related peptide promotes H3K9 acetylation and, consequently, H3K9 hyperacetylation promotes chronic neuropathic pain through inflammatory regulation and the production of pro‐inflammatory factors in the neural microenvironment [19, 20].

It is known that the ING5 protein, also found in the Giant Cell Lesions, interacts with the MOZ/Morf protein complex to promote acetylation on histone H3. Overexpression of ING5 can increase osteoclast differentiation through the NF‐кB signaling pathway. The accumulation of this protein in the cytoplasm is associated with aggressive characteristics (e.g., invasion and migration) and the activation of autophagy in gastric cancer cells [21, 22, 23, 24]. Additionally, ING5's upregulation may reflect the increase in the rate of differentiation and osteoclastic activity since our results showed ING5 overexpression in MGC and CMGC of aggressive Giant Cell Lesions, as well as the association of this protein with growth, tooth displacement, root resorption, and cortical bone perforation features in aggressive CGCG.

Cell cannibalism has increasingly been recognized as a key feature of cellular adaptation and survival in pathological environments [6]. The expression of H3K9ac and ING5 in cannibalistic multinucleated giant cells (CMGCs) may indicate a synergistic interaction where this epigenetic modification promotes metabolic and phenotypic changes associated with increased aggressiveness. In this context, H3K9 hyperacetylation could be acting to support the survival and boost the osteoclastic activity of MGCs and CMGCs by upregulating genes involved in proliferation, differentiation, stress responses, and osteoclastic functions, while ING5 further reinforces these effects by promoting more H3K9 acetylation in the microenvironment of aggressive Giant Cell Lesions [9, 10, 11, 17, 18, 24].

Clinically, measuring H3K9ac and ING5 levels in these lesions might improve diagnostic and prognostic accuracy, as differential immunoprofiling of these markers can enhance diagnostic precision and guide treatment planning. Furthermore, increased expression or altered localization of H3K9ac and ING5, particularly in MGCs and CMGCs, may signal elevated osteoclastic activity and be associated with clinical features such as growth, as well as with tooth and bone tissue destruction. This information could be valuable for stratifying clinical behavior and monitoring therapeutic responses. However, to validate their clinical relevance, future research should aim to investigate H3K9ac and ING5 as potential biomarkers by correlating expression patterns with long‐term patient outcomes.

Our results provide substantial evidence that epigenetic events contribute to the pathogenesis of Giant Cell Lesions and suggest that H3K9ac plays a role in osteoclast differentiation and activity in aggressive lesions. Management of CGCG varies according to the clinical behavior and size of the lesions. The treatment ranges from invasive surgical approaches to conservative therapies such as intralesional corticoids and monoclonal antibodies [15]. From a therapeutic point of view, our findings also suggest that epigenetic drugs may serve as therapeutic agents. However, this possibility remains to be explored and validated through future functional studies and longitudinal clinical investigations assessing recurrence and therapeutic response.

In this context, it has been demonstrated that epigenetic modulators such as Sodium phenylbutyrate, Trichostatin, Vorinostat, and Entinostat can suppress osteoclast differentiation [25, 26], while valproic acid has shown bone tissue repair effects [27]. Other modulators, such as Romidepsin, Panobinostat, and Dacinostat, have been identified as growth inhibition strategies for treating GCTB [28]. From this perspective, there is a need to investigate the use of epigenetic drugs for CGCG treatment and patient rehabilitation before they are used in clinical settings.

While these findings offer valuable insights into H3K9 hyperacetylation and the aggressiveness of Giant Cell Lesions, a limitation of this study is the absence of clinical data on CGCG recurrence. Such data would enhance understanding of how H3K9 hyperacetylation influences clinical outcomes, including recurrence rates or post‐treatment course. Additionally, despite the computer‐assisted analysis, reliance on immunohistochemical assessment by a single examiner may be another limitation, as ideally it should involve calibration and evaluation by multiple examiners. These limitations highlight the need for future research incorporating multi‐observer validation and extended clinical follow‐up to determine this marker's potential to improve diagnostic and prognostic accuracy. Despite these limitations, our results provide substantial evidence that epigenetic mechanisms, particularly H3K9 hyperacetylation, may play a role in osteoclast differentiation and activity in aggressive Central Giant Cell Granuloma.

## Author Contributions

**Caio César da Silva Barros:** conceptualization, investigation, funding acquisition, writing – original draft, methodology, validation, writing – review and editing, formal analysis, data curation. **Éricka Janine Dantas da Silveira:** conceptualization, investigation, funding acquisition, writing – original draft, methodology, writing – review and editing, formal analysis, project administration, supervision, data curation. **Cristiane Helena Squarize:** conceptualization, writing – original draft, methodology, writing – review and editing, formal analysis, supervision, data curation. **Rogerio Moraes Castilho:** conceptualization, writing – original draft, formal analysis, writing – review and editing, methodology.

## Funding

This work was supported by the Coordenação de Aperfeiçoamento de Pessoal de Nível Superior (Grant No. 88887.568762/2020‐00).

## Ethics Statement

This work was approved by the review board and Research Ethics Committee of the Federal University of Rio Grande do Norte (approval #5,632,269) and adheres to current recognized ethical guidelines.

## Conflicts of Interest

The authors declare no conflicts of interest.

## Supporting information


**Figure S1:** Spearman correlation matrix. Correlation between Histone H3 lysine 9 acetylation (H3K9ac) and ING5 immunoexpression in mononuclear cells (MC), multinucleated giant cells (MGC), and cannibal multinucleated giant cells (CMGC) of (A) non‐aggressive and (B) aggressive central giant cell granuloma. As shown in the color key, green and red denote positive and negative correlations, respectively (*p*‐values: *p ≤ 0.05; **p ≤ 0.01; ***p ≤ 0.001; ****p ≤ 0.0001).

## Data Availability

The data that support the findings of this study are available from the corresponding author upon reasonable request.
