# Supplementary figures and images for: Histone 3 Hyperacetylation and the Aggressive Behavior of Giant Cell Lesions
Source: Cancer Med. 2026 Apr 5;15(4):e71785. doi: 10.1002/cam4.71785 (PMC13052106; doi:10.1002/cam4.71785)

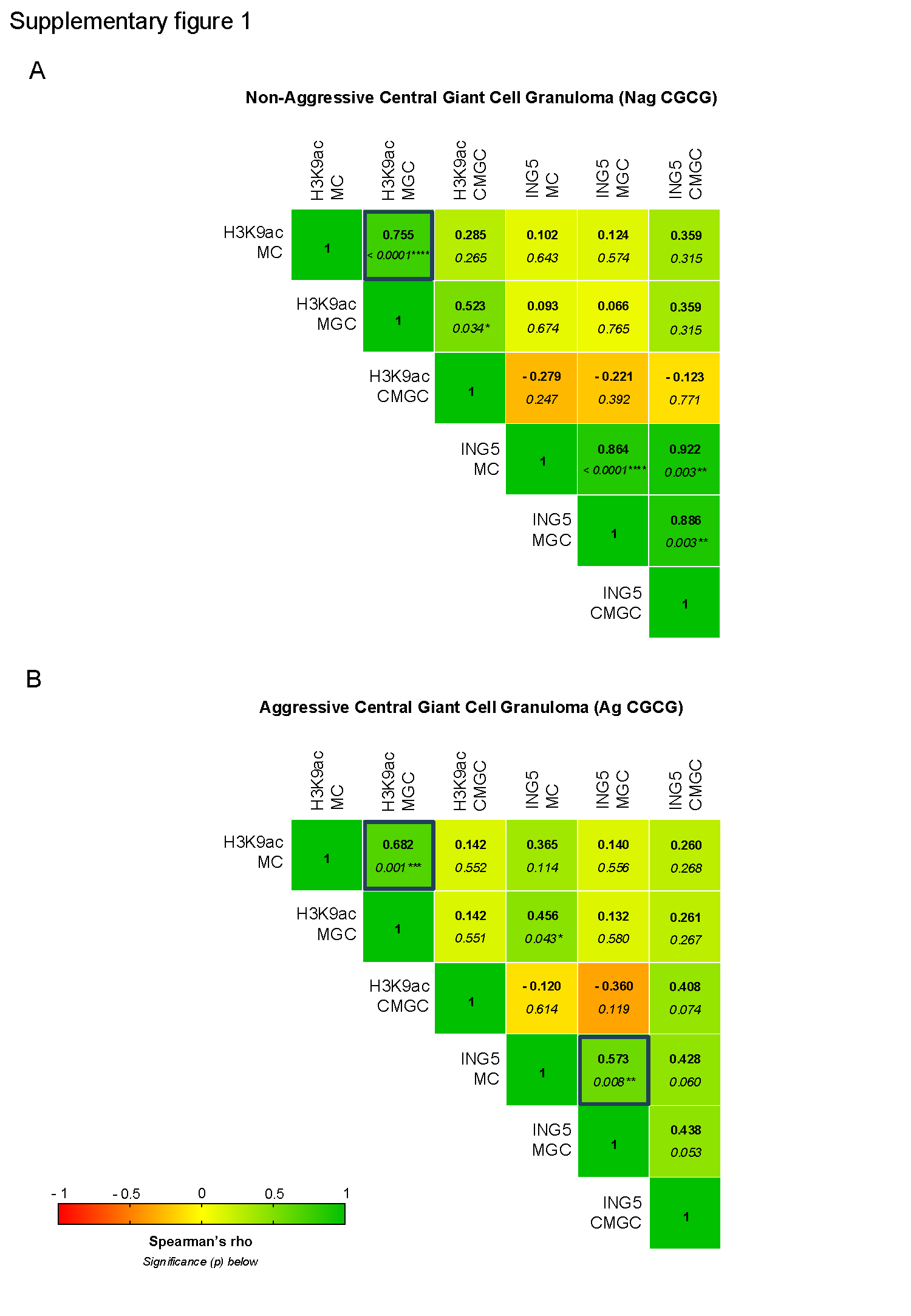

Supplement: Supplementary file 1 — Figure S1: Spearman correlation matrix. Correlation between Histone H3 lysine 9 acetylation (H3K9ac) and ING5 immunoexpression in mononuclear cells (MC), multinucleated giant cells (MGC), and cannibal multinucleated giant cells (CMGC) of (A) non‐aggressive and (B) aggressive central giant cell granuloma. As shown in the color key, green and red denote positive and negative correlations, respectively (p‐values: *p ≤ 0.05; **p ≤ 0.01; ***p ≤ 0.001; ****p ≤ 0.0001). [file CAM4-15-e71785-s001.tiff]
